# Extracellular Guanosine 5′-Triphosphate Induces Human Muscle Satellite Cells to Release Exosomes Stuffed With Guanosine

**DOI:** 10.3389/fphar.2018.00152

**Published:** 2018-03-16

**Authors:** Tiziana Pietrangelo, Ester S. Di Filippo, Marcello Locatelli, Francesco Piacenza, Marco Farina, Eleonora Pavoni, Andrea Di Donato, Denise Innosa, Mauro Provinciali, Stefania Fulle

**Affiliations:** ^1^Dipartimento Neuroscienze Imaging and Scienze Cliniche, Università degli Studi “G. d’Annunzio” Chieti-Pescara, Chieti, Italy; ^2^Dipartimento di Farmacia, Università degli Studi “G. d’Annunzio” Chieti-Pescara, Chieti, Italy; ^3^IRCCS-Istituto Nazionale di Riposo e Cura per Anziani, Polo Scientifico e Tecnologico, Centro di Tecnologie Avanzate nell’Invecchiamento, Ancona, Italy; ^4^Dipartimento di Ingegneria dell’Informazione, Università Politecnica delle Marche, Ancona, Italy; ^5^Harvard John A. Paulson School of Engineering and Applied Sciences, Harvard University, Cambridge, MA, United States; ^6^Facoltà di Bioscienze e Tecnologie Agro-Alimentari e Ambientali, Università di Teramo, Teramo, Italy

**Keywords:** satellite cells, guanosine, guanosine 5′-triphosphate, myomiRNA, exosomes, skeletal muscle regeneration

## Abstract

The extracellular guanosine 5′-triphosphate, GTP, has been demonstrated to be an enhancer of myogenic cell differentiation in a murine cell line, not yet in human muscle cells. Our hypothesis was that GTP could influence also human skeletal muscle regeneration, specifically in the first phases. We tested GTP stimulus on human muscle precursor cells established in culture by human satellite cells derived from Vastus Lateralis of three young male. Our data show that extracellular GTP (a) up-regulated miRNA (specifically miR133a and miR133b) and myogenic regulator factor and (b) induces human myogenic precursor cells to release exosomes stuffed with guanosine based molecules (mainly guanosine) in the extracellular milieu. We think that probably these exosomes could be addressed to influence by means of their content (mainly guanosine) in paracrine or autocrine manner the surrounding cells and/or at distance other muscles or tissues.

## Introduction

It has been extensively demonstrated that extracellular nucleotides are important intercellular messengers that mediate their signals through activation of P2 purinoceptors on the cell membrane (Burnstock, 2017). However, to date no indication exists about other ways of nucleotide signaling. In the last years, the communication by extracellular vesicles in muscle has received a great attention due to their importance as intercellular mediators bearing proteins, lipids, RNAs, and miRNAs through the entire body (Murphy et al., 2017). Exosomes are secreted by cells and released in the extracellular *melieu* where they could keep in contact with the surrounding cells or could be captured into the flowing blood or the lymph and addressed to the entire body. In this manner, the exosomes, and specifically their content, keep in contact different tissues influencing them as physiologycal mediators and/or pathological inductors. The adult skeletal muscle stem cells, satellite cells, provide a high degree of plasticity to muscles. During youth, the activated satellite cells form new myofiber and make hypertrophic the existing ones; during adulthood they compensate for muscle turnover due to daily wear (Yin et al., 2013). Recently, it has been demonstrated that satellite cells are involved in the cellular turnover in response to the ingestion of protein dense foods and exercise (Burd and De Lisio, 2017). Moreover, the skeletal muscle regeneration greatly relies on the dynamic interplay between satellite cells and their cellular niche. The 3D culture model of fibers has been demonstrated to be physiologically relevant to study *in vitro* this niche and the signals present in it (Said et al., 2017; Kasper et al., 2018). The interest on studying skeletal muscle regeneration is exponentially increasing due to also its implication in nutritional aspects, exercise and healthy life. Many papers and reviews outline the role of satellite cells in muscle homeostasis during exercise stimulus or in pathological conditions, when the relative roles of intrinsic vs. extrinsic factors contribute to satellite cell dysfunction (Pietrangelo et al., 2015; Cornelison, 2018).

The pools of satellite cells are activated to proliferate under myogenic regulator factors that control both an initial boost of proliferation and differentiation. These phases influence and in turn are influenced both by myogenic regulator factors as Myogenin and by myo-microRNA, specifically miR-1, miR133a/b, and miR-206 (Drummond et al., 2011; Di Filippo et al., 2017). To date, the study of the role of nucleotide signaling in skeletal muscle regeneration is underestimated. We previously demonstrated that extracellular GTP is able to bind specific sites on plasma membrane and it participates in the early phases of myogenic differentiation process of murine C2C12 cells, modifying the plasma membrane potential toward hyperpolarized state and accelerating the proliferative boost (Pietrangelo et al., 2002, 2006a; Mancinelli et al., 2012). To date it is not known whether extracellular GTP also affects the human satellite cells in the phases of differentiation process. We therefore, hypothesize that extracellular GTP could influence the satellite stem cells via both typical signal transduction (binding on receptors) and/or by release of exosomes stuffed with guanosine based molecules.

## Materials and Methods

### Cell Cultures

The Vastus Lateralis skeletal muscle biopsies were obtained by means of tiny percutaneous needle-biopsy (Pietrangelo et al., 2011).

The subjects who volunteered to have Vastus lateralis needle-biopsy to collect satellite cells provided written informed consent. The study was conducted according to the Helsinki Declaration (as amended in 2000) and approved by the Ethics Committee of the Università degli Studi “G. d’Annunzio” Chieti-Pescara, Italy (protocol no. 773 COET).

To obtain satellite sells, the skeletal muscle was processed according to the method described in Mancinelli et al. (2016). Human satellite cells were isolated from three young male volunteers (22.7 ± 2.1 years; *n* = 3), were grown as human myogenic precursor cells (MPCs) at 37°C in 5% CO_2_ humidity, in Growth Medium (GM): HAM’s Nutrient Mixture F10 medium (Euroclone, Milan, Italy, product #ECB7503SL) supplemented with 20% fetal bovine serum (FBS; Hyclone, Euroclone, product #SH30070.03), 100 U/ml penicillin, 100 μg/ml streptomycin, 50 μg/ml gentamycin (Euroclone, product #ECM0011B) and 1% glutamax. The MPCs were plated at a confluence of 15,000 cells/cm^2^ and maintained for 2 days in Growth Medium. After 2 days in GM, the cells were cultivated with fresh GM for additional 24 h (CTR-undiff) or stimulated by addition of 500 μM GTP for the following 24 h (GTP-undiff). Differentiation was induced by replacing the GM with the Differentiation Medium (DM) on cells plated on growth condition 3 days before. The differentiating cells were regularly cultivated for 24 h (CTR-diff) or stimulated by addition of 500 μM GTP for 24 h (GTP-diff). DM is composed of DMEM high Glucose (Euroclone, product #ECB7501L) supplemented with 5% heat-inactivated Horse Serum (HS) (56°C, 30 min) (HS; Euroclone, product #ECS0091L), 50 μg/ml gentamycin (Euroclone, product #ECM0011B), 10 μg/ml insulin (Sigma–Aldrich, Milan, Italy, product #I-0516) and 100 μg/ml of apo-transferrin (Sigma–Aldrich, product #T2036).

### Immunocytochemistry and Flow Cytometric Analysis

To estimate the myogenic purity of our cultures, we performed immunocytochemistry assays using Desmin as a marker. Desmin is a cytoskeletal intermediate filament protein early expressed in myogenic cell populations and not in fibroblasts. The myogenic purity of each MPC culture was estimated using Desmin antibody D33 as marker (DAKO, product #M0760). Specific antibody binding was revealed using the streptavidin biotinylated peroxidase visualization reagent (StreptABComplex DAKO). The percentage of desmin positive myoblasts was estimated as the number of positive cells vs. the total number of cells. Furthermore, human cell cultures were analyzed by flow cytometric analysis for surface myogenic markers using CD56 Mouse monoclonal antibody (Abcam, Cambridge, United Kingdom) and 5.1H11 policlonal antibody [Developmental Studies Hybridoma Bank (DSHB), Iowa City, IA, United States] (Di Filippo et al., 2016).

### Gene Expression Profile

The RNA was extracted from the human MPCs in undifferentiated cells (CTR-undiff) and with GTP stimulation for 24 h (GTP-undiff), and also in differentiation medium for 24 h (CTR-diff) and with GTP stimulation 24 h (GTP-diff) using PureLink RNA mini kit (Life Technologies). After RNA quantification using a NanoDrop^TM^ spectrophotometer, 500 ng was processed to obtain cDNA by High Capacity cDNA Reverse Transcription (#4368814; Applied Biosystems). We evaluated by quantitative real-time PCR the following genes: paired box (Pax) 7 (#4331182, Hs00242962_m1); myogenic differentiation (MyoD) 1 (#4331182, Hs00159528_m1), myogenin (#4331182, Hs 01072232GEXper-design_m1). Glyceraldehyde-3-phosphate dehydrogenase (GAPDH; #4331182, Hs99999905_m1) was used as the internal control, and the data are shown as difference in cycle threshold (ΔCt). An Applied Biosystems Prism 7900HT Sequence Detection System was used, with the Sequence Detector Software (SDS version 2.0; Applied Biosystems) (Di Filippo et al., 2017).

### GTP-Dependent microRNA Expression Profile

To perform the microRNA analysis, small RNA extractions from the human MPCs in undifferentiated cells (CTR-undiff) and with GTP stimulation for 24 h (GTP-undiff), and also in differentiation medium for 24 h (CTR-diff) and with GTP stimulation 24 h (GTP-diff) was isolated using PureLink miRNA Isolation kits (Invitrogen, Life Technologies) following the manufacturer instructions and according to the procedure of Mancinelli et al. (2016). We also evaluated the following microRNA, called myomiRs: has-miR-1 (#0 02222); has-miR-206 (#00 0510); has-miR-133b (#00 2247); has-miR-16-5p (#00 0391). miR-16 was used as an internal control. The relative quantification of the miRNA targets was carried out using the ΔCt formula (Ct_miRNAofinterest_ e Ct_miR-16_), according to the Ct method.

### Exosome Isolation and Purification From Cellular Media

Exosomes have been purified from both GM and DM and from media conditioned with cell culture as CTR-undiff and -diff and GTP-undiff and -diff samples following the method published by Thery et al. (2006). In detail, after a first centrifugation 10 min at 300 *g*, 4°C to pellet cells, the conditioned-cell culture media was centrifuged 20 min at 2,000 *g*, 4°C in order to remove dead cells. The supernatant was then centrifuged 30 min at 10,000 *g*, 4°C to remove cell debris. The resulting supernatant was centrifuged 70 min at 1,00,000 *g*, 4°C to collect the pellet containing exosomes. A final step at the same speed was performed to obtain purified exosomes. The resulting pellet was resuspended in 150 μL of PBS. All the centrifugation steps were performed with the Sorvall Discovery 90SE Centrifuge.

### Western Blotting for Exosome Identification

Exosomes were lysed with Exosome Resuspension Buffer (Thermo Fisher Scientific, Cat # 4478545), and denatured for 5 min at 75°C, loaded onto Bolt^TM^ 4–12% Bis-Tris Plus Gels (invitrogen REF NW04120BOX), run at 150 V for 35 min and transferred to nitrocellulose membrane with iBlot 2 Dry Blotting System (Thermo Fisher Scientific, Cat # IB21001). Membrane was blocked 1 h at RT with 5% Skim-milk (Sigma–Aldrich) in TBS buffer supplemented with 0.1% Tween-20 (Sigma–Aldrich), the membrane was incubated overnight at 4°C with primary antibodies, as follows: CD63 (MX-49.129.5, sc-5275, Santa Cruz Biotechnology, Inc.) at 1:200, CD81 (1.3.3.22, sc-7637, Santa Cruz Biotechnology, Inc.) at 1:200. After incubation with anti-mouse IgG, HRP-linked Antibody (Cell Signaling Technology) at 1:5000 for 1 h at RT. Immunodetection was performed with the LiteAblot PLUS enhanced chemiluminiscent substrate (EuroClone). Images were taken at UVITEC machine (Cambridge).

### Guanosine-Based Molecules Determination by HPLC-PDA

For the guanosine based-molecules separation was used a mobile phase composed by phosphate buffer (pH 7, concentration 40 mM) as solvent A and acetonitrile as solvent B at a flow rate of 1 mL/min. The column used for analytes resolution is the XTIMATE C18 (4.6 mm × 250 mm, 5 μm, Welch, Shanghai, China) and the system was thermostated at 25°C (±1°C). The gradient profile used to resolve the four compounds was reported in **Table 1**. The samples (20 μL) were directly injected into the HPLC-PDA system after a preliminary centrifuged at 12,000 rpm and without further pre-treatment. All the analytes were detected and quantified at their corresponding maximum wavelengths, such as 256 nm for GTP, GDP, GMP, and 259 nm for guanosine (**Table 2**).

**Table 1:** Gradient elution profile used for the four analytes.

Time (min)	Flow rate (mL/min)	%A	%B
0	1 mL/min	100	0
5		80	20
7		80	20
7.10		100	0
18		100	0

**Table 2:** Calibration parameters for the four analytes.

Analytes	Retention times (min)	Wavelengths (nm)	LOQ (LOD) (μg/mL)	Linearity (μg/mL)	*r*^2^
GTP	3.34 ± 0.03	256	0.1 (0.03)	0.1–5	0.9987
GDP	3.64 ± 0.05	256	0.1 (0.03)	0.1–5	0.9996
GMP	4.46 ± 0.08	256	0.1 (0.03)	0.1–5	0.9997
Guanosine	6.03 ± 0.06	259	0.1 (0.03)	0.1–5	0.9980

*LOD, limit of detection; LOQ, limit of quantification.*

During the method development, several parameters have been tested and optimized, such as column, mobile phase composition and temperature. The first parameter evaluated was the mobile phase composition, which was optimal when consisting of phosphate buffer (pH 7, concentration 40 mM) and acetonitrile. The column used for analytes resolution is the XTIMATE C18 (4.6 mm × 250 mm, 5 μm, Welch, Shanghai, China). Among the evaluated parameters, however, it is also necessary to remember the type of elution chosen. Firstly, was tested isocratic elution with PBS and AcN in ratio 97:3 in order to retain the more hydrophobic compounds and to reduce the total run-time. Isocratic conditions were tested in order to avoid transferability drawbacks. Unfortunately, these conditions do not allow to fully resolving the analyte peaks and it was therefore necessary to proceed using gradient elution. Some gradient elution profiles were tested and the best conditions in terms of total run-time and peaks resolution were observed using the gradient reported in **Table 1** at 25°C.

**FIGURE 1:**
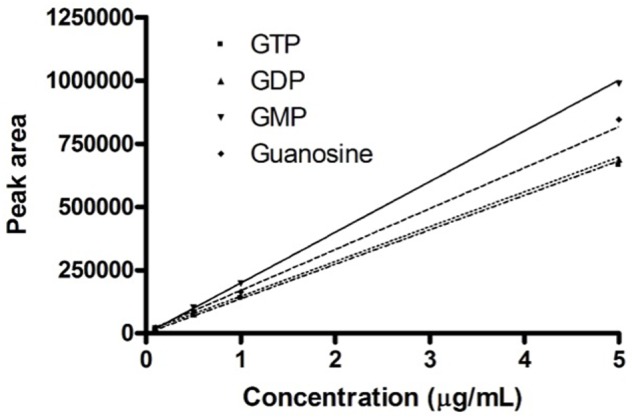
Calibration curves. Calibration curves obtained for the four guanosine-based analytes. The graph shows the concentrations of guanosine-based molecules and the relative peak area measured on the developed HPLC procedure.

In this range of linearity (**Figure 1**), the HPLC procedure fulfills the validation parameters as requested by International Guidelines (CDER and CVM Guidance for Industry, 2001; Taverniers et al., 2004; ICH, 2005).

Preliminary analysis of exosomes deriving from fresh media GM and DM, revealed the only presence of GDP as 0.10–0.12 ± 0.01 μg mL^-1^.

### Nanometric Scanning of Exosomes by Atomic Force Microscopy

The exosomes derived from media conditioned by MPCs in presence or not of extracellular GTP were scanned to analyze their physical properties. Experimental data were obtained using the NT-MDT Solver Pro P-47 AF. The measurements were collected in semi-contact mode by using a probe with a resonant frequency of 130 kHz and a spring constant of 4.4 N/m (HA_NC ETALON, NT-MDT). The images were obtained by plotting the error signal (mag) over 256 × 256 (x,y) positions; the mag signal is related to the oscillation amplitude of the cantilever and corresponds to the error signal of the feedback loop when working in semi-contact mode; the error signal is used for a more detailed information on the surface topography. Particle size distribution was performed with Nova Software in 1 μm × 1 μm scan area and by using a threshold of 10 nm. A statistical *t*-test has been used to evaluate the significance between the diameter distribution (*p* < 0.05).

### Statistical Analysis

Data were expressed as means ± standard deviations. Statistical significance was set at *p* < 0.05 and was calculated using the unpaired Student’s *t*-test. Prism5 GraphPad software (Abacus Concepts GraphPad Software, San Diego, CA, United States).

## Results

### Human Myogenic Precursor Cells

**FIGURE 2:**
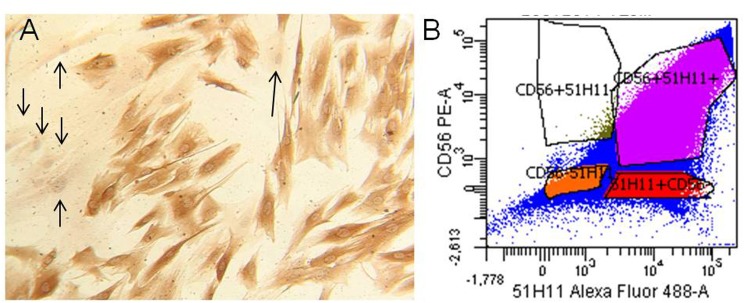
Human myogenic precursor cells (MPCs). Human MPCs characterization. **(A)** Representative example of Desmin-positive (brown) and Desmin-negative (arrows) cells revealed by biotin-streptavidin complex method. **(B)** Representative example of flow cytometric analysis for two surface myogenic markers (CD56 and 5.1H11). In the example the percentage of CD56+/5.1H11+ cells was 75.1% and CD56–/5.1H11– cell was 11.2%.

We obtained three different human MPC culture named #1 (85.31% desmin positivity), #2 (61.6% desmin positivity), and #3 (77.8% desmin positivity). The MPC cultures were analyzed by flow cytometric analysis for surface myogenic markers using the mouse monoclonal antibody CD56 (Abcam, Cambridge, United Kingdom) and policlonal antibody 5.1H11 [Developmental Studies Hybridoma Bank (DSHB), Iowa City, IA, United States] to confirm the percentage of myogenicity. The percentage of CD56^+^/5.1H11^+^ cells was 75.1% and those CD56^-^/5.1H11^-^ was 11.2% (**Figure 2**).

### Quantitative Gene Expression RT-PCR on Myogenic Regulatory Factors

**FIGURE 3:**
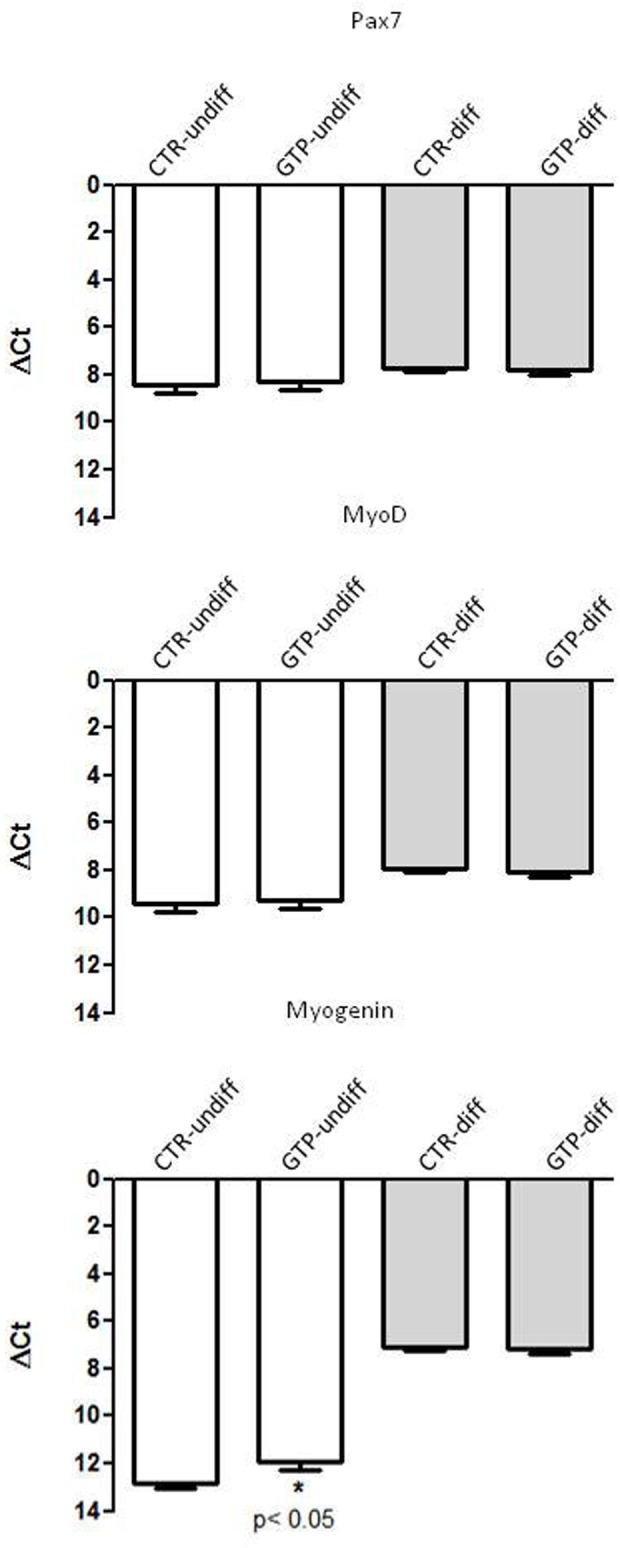
GTP-dependent Myogenic regulator factors in human MPCs. The graphs show the relative expression of Pax7, MyoD, and Myogenin genes in undifferentiated MPCs (CTR-undiff) and with GTP stimulation (GTP-undiff) and in MPCs after 24 h of differentiation medium (CTR-diff) and with GTP stimulation (GTP-diff). The data are as mean and SD for *n* = 3 independent experiments, each performed in triplicate.

The most important myogenic regulatory factors such as Pax7, MyoD, and Myogenin were assessed in our samples in order to investigate their involvement with GTP stimulation. Pax7 and MyoD1 did not change their expression in our samples, neither with respect to undifferentiated and 24-h differentiation (**Figure 3**). Myogenin was up-regulated after 24 h of differentiation stimulation both in CTR-diff and GTP-diff compared to CTR-undiff and GTP-undiff. Strikingly, we found Myogenin up-regulated after 24 h of 500 μM GTP stimulation in growth medium (GTP-undiff compared to CTR-undiff), but no differences were found in 24-h differentiated samples between CTR-diff and GTP-diff (**Figure 3**).

### GTP-Induced microRNA Modulation

**FIGURE 4:**
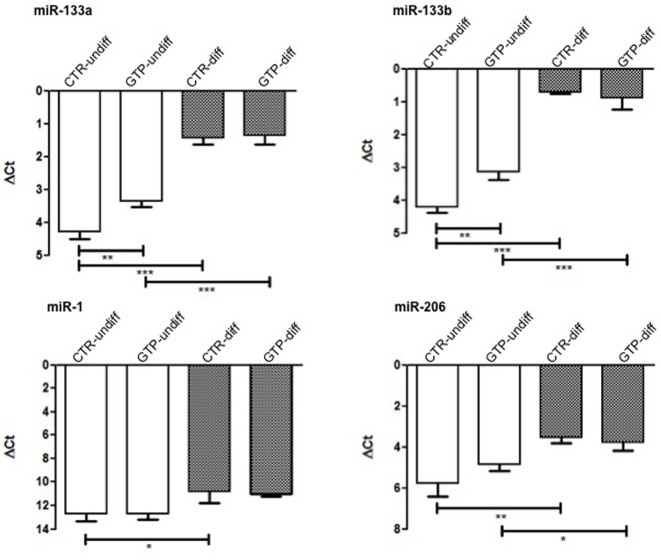
microRNA expression profile in human MPC cells after GTP stimulation. The graphs show the relative expression of miR-133a, miR-133b, miR-1, and miR-206 both in myoblasts (CTR-undiff), in cells differentiated for 24 h in DM (CTR-diff) and in 500 μM GTP-stimulated myoblasts and differentiating cells (GTP-undiff and GTP-diff, respectively). Mean ± SD, *n* = 3 independent experiments, each performed in triplicate. ^∗^*p* < 0.05, ^∗∗^*p* < 0.005, ^∗∗∗^*p* < 0.0001.

miR-133a and miR-133b were significantly up-regulated after 24 h of 500 μM GTP stimulation of myoblasts (GTP-undiff) compared to control (CTR-undiff). Furthermore, these microRNAs were found highly up-regulated in differentiation media both in CTR-diff and in GTP-diff. Also miR-1, that in myoblasts (CTR-undiff; GTP-undiff) does not change its expression, in differentiative condition, was slightly up-regulated both on CTR-diff and GTP-diff both at the same extent. Similarly, miR-206 was significant up-regulated after 24 h of differentiation stimulation both CTR-diff and GTP-diff compared to CTR-undiff and GTP-undiff, but no differences were found between CTR-diff and GTP-diff (**Figure 4**).

### Nanometric Scanning of Exosomes by Atomic Force Microscopy

**FIGURE 5:**
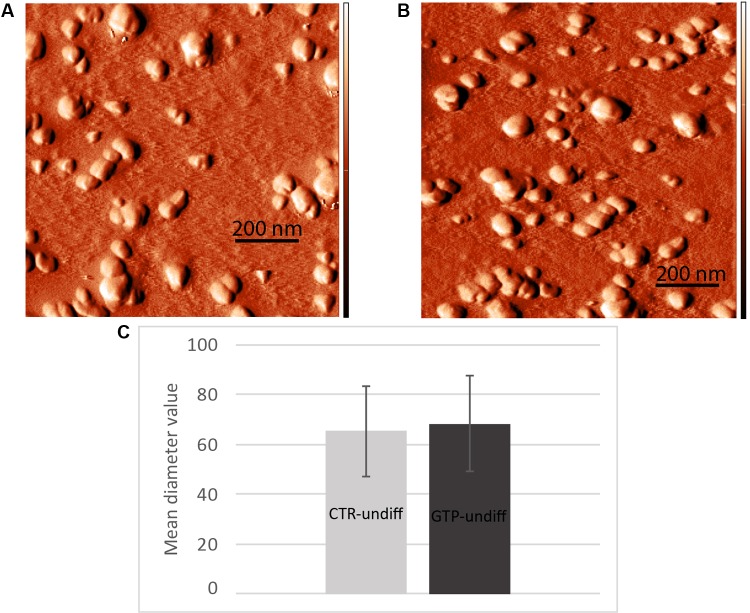
Nanometric scanning of exosomes by atomic force microscopy. The exosomes are rounded shaped structures showing dimensions of about 60–70 nm as mean diameter. Images represent an example of exosome topography acquired in semicontact scanning. AFM error signal images obtained in semi-contact mode [scan area 1 μm × 1 μm (x,y)] of undifferentiated control (**A**, CTR-undiff) and GTP-treated (**B**, GTP-undiff) exosomes. Mean diameter particle size distribution **(C)** evaluated in 1 μm × 1 μm scan area by using a threshold of 10 nm.

The images obtained by AFM (**Figures 5A,B**) show the typical round-shaped structure of exosomes with diameter values ranging between 30 and 100 nm. Specifically, the diameter values were 65 ± 18 and 68 ± 19 nm for CTR-undiff and GTP-undiff exosomes, respectively (**Figure 5C**). Both samples, control and GTP-treated exosomes, were analyzed in terms of diameter distribution and as can be seen from the images, the exosomes were not found to be significantly different (*p* < 0.05).

### Western Blotting for Exosome Identification

**FIGURE 6:**
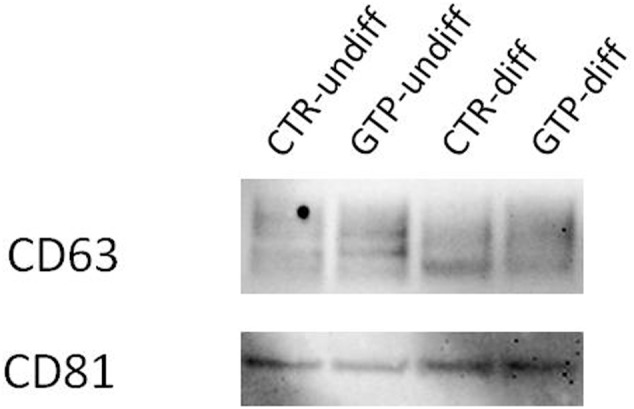
Western blotting for exosome identification. Representative image of Western blotting analysis of CD63 and CD81 expression on exosomes obtained by cell culture media named CTR-undiff and CTR–diff and GTP-undiff and GTP-diff samples. The image showed the presence of these proteins in the exosome samples we isolated by ultracentrifugation (see section “Materials and Methods”).

The western blot analysis using antibodies CD63 and CD81 (specifically expressed on exosomes), revealed the presence of these proteins on our isolated nanovesicles (**Figure 6**).

### HPLC-PDA Guanosine-Based Molecules Determination Into Exosomes

The exosomes obtained by both simple and cell-conditioned media with and without GTP were analyzed to detect guanosine-based molecules. The herein reported HPLC-PDA method allows quantify simultaneously the four guanosine-based molecules in exosomes extracted from culture media. The gradient elution allows separate and analyze these compounds better than isocratic condition, within an overall runtime of 18 min. The quantitative data are reported in **Table 3**.

**Table 3:** Concentration of guanosine-based molecules contained into exosome samples deriving from three different MPC culture of young subjects.

Samples	Concentration (μg mL^-1^)			
	GTP	GDP	GMP	Guanosine
#1 Exosomes from CTR-undiff	nd	nd	nd	nd
#1 Exosomes from CTR-diff	nd	nd	nd	nd
#1 Exosomes from GTP-undiff	nd	0.10 ± 0.01	nd	1.70 ± 0.18
#1 Exosomes from GTP-diff	nd	0.36 ± 0.04	nd	2.52 ± 0.25
#2 Exosomes from CTR-undiff	nd	nd	nd	nd
#2 Exosomes from CTR-diff	nd	nd	nd	nd
#2 Exosomes from GTP-undiff	nd	nd	0.15 ± 0.02	4.17 ± 0.35
#2 Exosomes from GTP-diff	0.33 ± 0.03	0.19 ± 0.01	nd	0.20 ± 0.02
#3 Exosomes from CTR-undiff	nd	nd	nd	nd
#3 Exosomes from CTR-diff	nd	nd	nd	nd
#3 Exosomes from GTP-undiff	nd	nd	nd	1.51 ± 0.12
#3 Exosomes from GTP-diff	0.16 ± 0.02	0.17 ± 0.02	nd	0.70 ± 0.05

*nd, not detected. #1, #2, #3 three different young volunteers from whom we obtained myogenic precursor cells. CTR-undiff = the exosomes obtained from the medium conditioned with the human MPC cells in proliferative state for3 days in GM. CTR-diff = the exosomes obtained from the medium conditioned with the human MPC cells in differentiative state for 24 h. GTP-undiff = the exosomes obtained from the medium conditioned with the human MPC cells in proliferative state for additional 24 h after 2 days of regular cultivation in GM. GTP-diff = the exosomes obtained from the medium conditioned with the human MPC cells in differentiative state in presence of 500 μM extracellular GTP for 24 h.*

## Discussion and Conclusion

We previously demonstrated that extracellular GTP binds its specific binding site on C2C12 myoblasts and triggers specific signal transduction that lead the myoblasts to increase the intracellular calcium and to hyperpolarize the plasma membrane (Pietrangelo et al., 2006a). However, GTP works also as intracellular signal, it triggers molecules for the G-coupled receptor signaling or its de-phosphorylation is instrumental for effector enzyme activation. In addition, GTP shows inhibitory effect at uncoupling proteins of the mitochondrial anion carrier protein family (Woyda-Ploszczyca and Jarmuszkiewicz, 2017). Low mitochondrial potential increases the inhibitory effect of GTP. In particular, GTP–GDP inhibits the proton leak mediated by these carriers across the inner mitochondrial membrane in lizard skeletal muscle mitochondria (Rey et al., 2008). In the cytoplasm GTP is present at mM level but also externally its effective concentration is in the order of 100s of micromolar (Azzu and Brand, 2010). Moreover, the cells are able to phosphorylate guanosine molecule to obtain GMP, GDP, and GTP and in turn they could be released. However, in the extracellular space, the ectonucleotidase binds and hydrolyze the GTP to GDP, GMP, and guanosine (Kovács et al., 2011). In this scenario, it is difficult to understand how guanosine-based molecules and particularly GTP could be secreted in the extracellular space and directed toward cell target, escaping the ectonucleotide degradation. The exosomes are small vesicles that protect and carry molecules to specific target. Our hypothesis that the GTP-incubated cells could produce some categories of exosomes stuffed with guanosine-based purines has been verified because we found mainly guanosine in the exosomes, but also GTP, GDP, and GMP even if in few samples. Specifically, the human MPCs that we stimulated with 500 μM extracellular GTP for 24 h, both in proliferative and differentiative conditions, secreted exosomes stuffed with guanosine in the range of 1.51–4.17 and 0.2–2.5 μg mL^-1^, respectively. This result suggests that extracellular GTP influences skeletal muscle exosome release in order to direct guanosine molecules via exosomes as both paracrine and endocrine transmission in the body. We think that these exosomes containing guanosine could influence surrounding cells in the niche of regenerating skeletal muscle and in addition could spread out in all other muscles of the body and other tissue via blood and/or lymphatic fluxes. The scenario we envisioned if that the extracellular free GTP could derive from a damaged skeletal fiber after injury or after vigorous muscle contractions following specific training and/or could be released physiologically during muscle growth. The extracellular free GTP stimulus in a specific fiber, could influence the entire skeletal tissue on the same muscle (Vastus Lateralis, for instance) or other skeletal muscles in the body via exosomes stuffed with guanosine, probably in a different manner with respect to the typical binding of ligand on a receptor. In our previous paper (Pietrangelo et al., 2006a) we demonstrated a specific free GTP effect in the first stage (the proliferative phase of myogenesis) of murine skeletal muscle regeneration. Here, we confirm that free GTP stimulates human MPCs in the proliferative boost phase of differentiation. In fact, extracellular GTP up-regulated the miR-133a and miR133b expression. These myo-miRNA are specifically linked to myogenic proliferation (La Rovere et al., 2014). The enhancer toward the differentiation process mediated by free extracellular GTP is confirmed by Myogenin gene upregulation during proliferation state. These results are in concordance with the fact that the pool of satellite cells is activated to proliferate under myogenic regulator factors that control both an initial boost of proliferation and a push of differentiation (Ceafalan et al., 2014). In conclusion, we think that free extracellular GTP elicited two effects: miRNA-myogenic regulator factor modulation and exosome stuffed with guanosine and guanosine-based molecules secretion. We think that these effects are sequential, in the sense that free GTP immediately influence MPC proliferation-differentiation step and in turn after 24 h induces secretion of exosomes contained-guanosine both in proliferative and differentiative conditions.

At the current state of the study, we do not have evidence for saying that exosome contained-guanosine based molecules could influence regeneration or differentiation. What we can hypothesize for future study is that these exosome stuffed with guanosine and guanosine based molecules (GMP, GDP, GTP) could be transferred into other tissues as neuronal and glial ones where exert specific effects (Gysbers et al., 2000; Pietrangelo et al., 2006b; Guarnieri et al., 2009; Giuliani et al., 2012, 2015; Mancinelli et al., 2014).

## Author Contributions

All authors listed have made a substantial, direct and intellectual contribution to the work, and approved it for publication.

## Conflict of Interest Statement

The authors declare that the research was conducted in the absence of any commercial or financial relationships that could be construed as a potential conflict of interest.
